# Can Male Fertility Be Improved Prior to Assisted Reproduction through The Control of Uncommonly Considered Factors?

**Published:** 2013-03-03

**Authors:** Daniel M. Campagne

**Affiliations:** Department of Personality, Evaluation and Psychological Treatment, Faculty of Psychology, UNED University, Juan del Rosal, Madrid, Spain

**Keywords:** Male Fertility, ART, Sperm, Environmental, Psychological, Lifestyle

## Abstract

Male factor infertility or subfertility is responsible for up to 50% of infertility cases. A considerable body of recent studies indicates that lifestyle as well as environmental and psychological factors can negatively affect male fertility, more than previously thought. These negative effects have been shown in many cases to be reversible. This review aims to provide a rationale for early clinical attention to these factors and presents a non-exhaustive evidence-based collection of primary relevant conditions and recommendations, specifically with a view to making first line diagnostics and recommendations. The presently available evidence suggests that considering the high cost, success rates, and possible side effects of assisted reproduction techniques (ART), such as *in vitro* fertilization (IVF) and intracytoplasmic sperm injection (ICSI), early efforts to improve male fertility appear to be an attainable and worthwhile primary goal.

A series of searches was conducted of Medline, Cochrane and related databases from November 14^th^, 2010 to January 26^th^, 2012 with the following keywords: male, fertility, infertility, sperm defects, IVF, ICSI, healthy habits, and lifestyle. Subsequent follow-up searches were performed for upcoming links. The total number of studies contemplated were 1265; of these, 296 studies were reviewed with criteria of relevance; the date of study or review; study sample size and study type; and publishing journal impact status. Data were abstracted based upon probable general clinical relevancy and use. Only a selection of the references has been reflected here because of space limitations. The main results obtained were evidence-supported indications as to the other causes of male infertility, their early detection, and treatment.

## Introduction

Causes of male infertility may be reversible and their treatment can benefit from early clinical verification and intervention. A selection of the newer published evidence is given here. In about 50% of cases, male factors play a role in a couple's infertility (1, 2). Medical science still has problems determining when and whether a male (sperm) problem is the primary or contributing cause for problems with fertility. Thus any factors that may affect sperm quality are relevant for any attempt at pregnancy; more so since more evidence points at an increased risk for birth defects in children conceived by *in vitro* fertilization (IVF) and intracytoplasmic sperm injection (ICSI) (3).

Professional ethics and good clinical procedure require that, before high-tech methods are considered, systemized efforts be employed to improve fertility and *ergo* sperm quality by changes in lifestyle and environmental and psychological factors (4). This “staircase-principle” has not yet been incorporated into the world’s main practice guidelines and is not always sufficiently applied in clinical practice.

Men react to infertility in their own particular way and study data should not be generalized between genders. “The information-seeking coping style was significantly correlated with infertility distress only among men (p<0.05)” (5). Thus, in order to motivate men to actively participate in attempts to resolve infertility issues, clear and concise information should be provided. This article cannot be all inclusive given the scope of the issue, but the intent is to provide a reasonable indication of the need to verify the presence of certain specific factors of male infertility, a subject that warrants further in-depth analysis and studies.

### Male infertility and undefined interactions

Men with a low sperm count can have children and those whose sperm counts are normal can be infertile. Many other aspects of sperm may influence infertility; rather, commenting on these in greater detail falls outside the scope of this article, which is not meant to analyze causes of male infertility, but to initially document the need for early attention to avoidable or remediable causes. The same goes for structural problems such as varicocele.

Sperm problems can be roughly divided into three categories: i. sperm morphology and defects, which include low motility or insufficient sperm (6); ii. DNA and chromosomal damage (7); and iii. surface markers or biochemical/acrosome problems (8). These categories possibly overlap, but studies affirm that they may be influenced by lifestyle, environmental, and psychological factors. For example, smoking reduces motility and sperm count, among its other effects. Thus when a smoker’s sperm is first analyzed, a low motility diagnosis and low sperm count can be forthcoming (9). Laboratory evidence has found smoking to also cause biochemical problems which prevent the sperm from binding to the ovum. Sperm carry a nicotinic cholinergic receptor, and it has been shown that chronic laboratory exposure to nicotine results in the binding of available nicotine to that receptor, causing a significant loss in the fertilizing capacity of sperm (10). Finally, smoking may produce DNA damage that is less easily diagnosed and has long-term negative effects (11). A 2011 study reviewing the frequency and distribution of disomy in sperm by multicolor-FISH analysis has indicated that lifestyle factors such as smoking are risk factors for sex chromosome abnormalities and disomies (12). A 2010 study reported that only 6% of smokers had normozoospermia, whilst 37% of nonalcoholic nonsmokers had normal sperm parameters (13). Smoking has a negative impact on semen variables and there is a correlation with increased sperm caspase-9, Smac/DIABLO and percent of DNA fragmentation, particularly in heavy smokers. Importantly, males who stopped smoking, after three months had a distinctive improvement in sperm concentration, fast spermatozoa, sperm vitality, percentage of spermatozoa that recovered after an enrichment technique and protein tyrosine phosphorylation. In this study no changes were observed in the number of germinal cells, sperm morphology and sperm DNA fragmentation. The authors concluded that “physicians should strongly advise their patients to quit smoking before undergoing medical treatment or assisted reproduction techniques (ART) to achieve pregnancy” (14).

Numerous other factors related to smoking have been identified but for our purpose the cited information is sufficient to consider the effects of smoking on male fertility as an example of an exogenous cause that can be identified and its effects reduced or (partly) reversed, thereby possibly restoring or achieving better levels of fertility.

### Main exogenous causes of male factor infertility

Available data suggest that at least 10% of male factor infertility is exogenous and reversible (15-17). The impacts of lifestyle, environmental, and psychological factors in the context of fertility treatments and in male factor infertility warrant close scrutiny as shown by the rapidly growing number of studies on this issue. In 2005, the American Society for Reproductive Medicine and the Canadian Fertility and Andrology Society dedicated annual meetings to this subject, after which the number of identified exogenous causes has grown considerably (18). In 2010, the European Society of Human Reproduction and Embryology (ESHRE) Task Force on Ethics and Law published a report on the influence of lifestyle factors on reproduction, which confirmed lifestyle to be “increasingly recognized as an outcome-determining factor in assisted reproduction, not only with regard to the cost-effectiveness but also in view of the balance of benefits and risks, including risks related to the welfare of the future child”. The report summarized evidence concerning the impact of obesity, tobacco smoking, and alcohol consumption on both natural and ART (19).

The following is indicative of some of the main evidence-supported factors that influence male fertility, which could benefit from early detection and treatment.

### Lifestyle factors

#### Acrylamide and glycidamide ingestion

Acrylamide, a xenobiotic compound present in certain foods (fried, baked, and starchy) and in the environment (tobacco smoke) is converted to its active metabolite glycidamide by the CYP2E1 enzyme. Large numbers of animal studies have shown this compound to be both carcinogenic and mutagenic. Reproductively it causes a decrease in sperm count, motility and morphology. Acrylamide produces clastogenic effects inducing disruption or breakage of chromosomes, whereas glycidamide has mutagenic effects. A number of possible protective measures against the effects of acrylamide include a more selective diet, probiotics, increased use of compounds known to decrease acrylamide production, and on a different scale, bioengineering of precursor foods such as potatoes (20).

#### Alcohol

Studies have identified the ingestion of alcohol as a testicular toxin that provokes abnormalities such as low sperm count and impaired sperm motility (21). These effects have been shown to be duration-dependent and reversible. Vicari et al. (22) have found that pregnancy was achieved three months after the cessation of alcohol consumption in the partner of an azoospermic patient which was secondary to heavy alcoholic intake. Sermondade et al. (23) have shown that stopping alcohol consumption led to a rapid, dramatic improvement in semen characteristics (azoospermia). In this study, strictly normal semen parameters were observed after three months. These findings confirmed several earlier studies. Alcohol, body mass index (BMI), other eating and social factors have been found to be detrimental to different aspects of male fertility according to another study in 2011 on 250 cases (24).

#### Bicycling (excessive)

Contrary to earlier indications, Wise et al. (25) found no significant influence of physical activity on overall sperm parameters, except for a negative influence from bicycling for more than five hours a week which was associated with low sperm concentration [odds ratio (OR): 1.92, 95% confidence interval (CI): 1.03-3.56] and low total motile sperm (TMS; OR: 2.05, 95% CI: 1.19-3.56). These associations did not vary appreciably by age, BMI, or history of male factor infertility.

#### Hot baths

Jung and Schuppe (26) reviewed the evidence on scrotal temperature and fertility. Studies that addressed professional exposure to high temperatures delivered conflicting results concerning fertility parameters. However, contraception via genital heat stress has been demonstrated using hot sitting baths or insulating suspensors. In a small study, wet heat exposure was a potentially reversible cause of low semen quality in infertile men, and scrotal cooling was found efficient in improving semen quality (27).

#### Recreational drug use

Anabolic-androgenic steroids, marijuana, cocaine, methamphetamines, and opioid narcotics have been demonstrated to be detrimental to male fertility. The hypothalamic-pituitary-testicular axis, sperm function, and testicular structure are adversely affected (28). Badawy et al.(29) have shown the two main active cannabinoids of the marijuana plant, delta-8 and 9-THC, to be potent inhibitors of mitochondrial oxygen consumption in human sperm, thus affecting sperm respiration and reducing fertility. Delta 9-THC has been found to increase ejaculation problems, reduce sperm count and motility, and generate loss of libido and impotence in men (30). Most adverse effects from drugs and medications have been shown to be reversed by discontinuing use of the offending agents (31).

#### Physical stress/exertion

Vigorexia, even in mild forms, affects fertility both through a higher incidence of structural problems such as varicocele and through sperm damage. After 24 weeks of exercise, those who performed high intensity exercise demonstrated significant declines in their semen parameters compared to those who exercised at a moderate intensity (p=0.03) (32).

#### Smoking tobacco (active and passive)

Zitzmann et al. (33) calculated the OR for treatment failure with paternal smoking compared to non smoking to be 2.65 for IVF and 2.95 for ICSI. Venners et al. (34) found a 77% increase in the OR for early pregnancy loss with paternal heavy smokers compared to “light” (<20/day) smokers. Marchetti et al. found that exposure to cigarette smoke (passive smoking) induced mutations at an expanded simple tandem repeat locus (Ms6-hm) in mouse sperm. However, it did not provoke genetic damage in somatic cells thus indicating that, as with acrylamide, the reproductive consequences of parent passive smoking affect future generations (35). Although recent reviews cite ample evidence (36, 37), the effects of smoking on male fertility warrant a specific in-depth review.

#### Vaginal lubricants and ultrasound gels

Agarwal et al. (38) have researched different types of vaginal lubricants detrimental to sperm. In their study, percentage motility did not differ significantly between controls and the U.S. brand Pre-Seed, whereas FemGlide, Replens, and Astroglide lubricants demonstrated a significant decrease in motility. A significant decline in sperm chromatin quality occurred with FemGlide and K-Y Jelly. Some lubricants and gels have been incorrectly labeled as “non-spermicidal”. A 2011 study has confirmed these results (39).

### Dietary factors

#### Body mass index (BMI)

Obesity leads to lower testosterone levels and other endocrine abnormalities are the usual consequence of obesity in conjunction with a higher scrotal temperature and higher incidence of erectile dysfunction. Weight reduction can improve the chances of natural conception according to the ESHRE Task Force (40). A 2009 Harvard Medical School study found that, contrary to earlier human and animal studies and despite major differences in reproductive hormone levels with increasing body weight, only extreme levels of obesity negatively influence male reproductive potential (41). This coincides with the findings of a 2011 Danish cohort study on the effects of weight reduction in severely obese (BMI>33) males which established an association between obesity and poor semen quality. However a 14-week (15%) weight loss led to improvements in total sperm count, semen volume, testosterone, sex hormone-binding globulin (SHBG) and anti-Müllerian hormone (AMH). The study group that lost more weight had a statistically significant increase in total sperm count and normal sperm morphology (42). Apart from natural weight loss, several therapeutic weight loss interventions are available, including minimally invasive surgical procedures (43).

#### Dietary supplements

Attempts at self-tuning health increasingly include dietary supplements which are often consumed without previous medical advice or control. Many supplements will probably not affect male fertility unless taken in high doses; some may be beneficial, whereas others are possibly harmful. In a prospective, randomized double-blind placebocontrolled trial (n=60), an antioxidant supplement has been shown to cause a statistically significant improvement in viable pregnancy rate (38.5%) compared to the control group (16%) (44). Omega- 3 polyunsaturated fatty acid supplementation has been found to improve the semen profile of infertile men diagnosed with idiopathic oligoasthenoteratospermia according to a recent doubleblind, placebo-controlled, randomized study (45). The administration of coenzyme Q10 was found to increase both ubiquinone and ubiquinol levels in semen and effective in improving sperm kinetic features in patients affected by idiopathic asthenozoospermia (46). Increasing evidence has supported the importance of adequate vitamins F and C, selenium, and zinc levels (47).

#### Hormones and hormone disruptors (endocrinedisrupting compounds, EDC)

Although many factors may affect male hormone levels, these do not always produce infertility. The cumulative effects of various low-dose exposures to endocrine disruptors in our environment produce case-dependent adverse effects in the male reproductive system (48). Semen quality may be the most sensitive marker of the total sum of adverse environmental exposures. The negative effects of anabolic steroids are documented (49), as well as those related to polychlorinated biphenyls, dioxins, polycyclic aromatic hydrocarbons, phthalates, bisphenol A, pesticides, alkylphenols and heavy metals (arsenic, cadmium, lead, and mercury) (50). Luccio-Camelo and Prins (51) have cited in vivo evidence for male reproductive tract disturbances that have arisen from these disruptors, which have the capacity to ligand the androgen receptor.

There are some indications that phyto-estrogens such as soy isoflavones may alter reproductive hormones, spermatogenesis, sperm capacitation and fertility. However, numerous other studies have neglected to observe the adverse effects on male reproductive physiology (52).

#### Oxidants

Free radicals are absorbed from the food or environment. Normal sperm function requires a certain level of free radicals, however excessive amounts of free radicals affect sperm function, fertilization and offspring health. Oxidative stress results when the balance is disrupted between free radicals and anti-oxidants. Oxidative stress is a well-established cause of male infertility, affecting up to 50% of infertile men. Reactive oxygen species (ROS) primarily cause infertility by impairing sperm motility and DNA integrity. Most clinical studies suggest that dietary antioxidant supplements are beneficial in terms of improving sperm function and DNA integrity (7). Oxidative stress tests include chemiluminescense, flow cytometry or the nitro blue tetrazolium (NBT) assay (53). In a 2009 study, oral antioxidant therapy has been shown to result in significant improvements in sperm DNA integrity (p=0.002) and protamine packaging (p<0.001), accompanied by a reduction in seminal ROS production (p=0.027) and apoptosis (p=0.004) (54). An earlier study (n=161) established that patients with normal seminal parameters and lower seminal leukocyte levels might benefit from this type of therapeutic intervention to lower ROS levels and improve semen quality (55). Oxidative stress is relevant for female fertility as well (56). Several herbal treatments have been found to mitigate oxidative stress. In infertile subjects, treatment with the herbal preparation Withania somnifera effectively reduced oxidative stress and improved the levels of semen quality indicators of total testosterone (T), luteinizing hormone (LH), follicle-stimulating hormone (FSH) and prolactin (PRL) (57). Several studies have found that Mucuna pruriens seed powder significantly improved sperm count and motility in infertile men. A 2011 proton nuclear magnetic resonance [(1) H NMR] spectroscopy study (n=180) found this substance to rectify perturbed alanine, citrate, glycerophosphocholine (GPC), histidine and phenylalanine content in seminal plasma (58).

### Environmental factors

#### Heavy metals

Cadmium, mercury, lead and arsenic levels are relevant for male fertility. Several well-designed studies with sufficient populations, appropriately adjusted for potential confounders, have noted their harmful effects on male reproduction. The evidence for the effects of low exposure was strongest for cadmium, lead, and mercury and less certain for arsenic. Conversely, traces of metals such as copper and manganese have been shown to be essential for sperm quality although excesses may cause adverse reproductive effects (59). Adequate levels of other metals such as zinc are beneficial for male fertility (60). Antioxidants and natural chelators play a role in the elimination of metals from the body. Occupational hazards, in general, need to be taken into account when considering male infertility (61).

#### (Soft) plastics and plasticizers

In many animal studies, phthalates have been shown to act as endocrine disruptors. In humans, males are most affected by the anti-androgenic action of several phthalates. A 2011 study of polyvinyl chloride pellet (PVC) factory workers in Taiwan was the first to demonstrate a link between di(2-ethylhexyl) phthalate (DEHP) concentration in ambient air and the adverse effects on sperm motility and chromatin DNA integrity (63, 63). Bisphenol A has shown to cause male infertility. Specific inhibitors and/or antagonists may be able to 'reverse' and/or 'block' the disruptive effects of toxicant-induced damage (64).

#### Pollution

There are numerous environmental toxicants whose negative effects on male fertility are often attributed to their combined interference (65).

#### Radiation

Exposure to radiation, both ionizing and nonionizing, has been shown to be a hormone disruptor. Cell phone radiation or radio-frequency electromagnetic fields (RF-EMFs) have become omnipresent factors. A 2006 Finnish study obtained the *in vitro* cell response to mobile phone radiation (900 MHz GSM signal) with two variants of a human endothelial cell line: EA.hy926 and EA.hy926v1. Gene expression changes were registered in three experiments using cDNA expression arrays and ten experiments examined protein expression changes. Gene and protein expression was shown to be altered in both cell lines after a one hour weight-specific exposure to mobile phone radiation (66). In a recent Canadian study, patients who used a cell phone showed significantly higher free testosterone and lower LH levels than those who did not, and sperm quality was negatively affected (67). A 2011 review has concluded that according to the total present data, human spermatozoa exposed to RF-EMF have decreased motility, morphometric abnormalities, and increased oxidative stress, whereas the use of mobile phones may decrease sperm concentration, motility (particularly rapid progressive motility), normal morphology, and viability. The abnormalities seemed to be directly related to the duration of mobile phone use (68). However, present evidence is inconclusive. Mobile phones produce radiation which may affect male fertility. In a 2011 prospective *in vitro* study (n=29), Avendaño et al. (69) found that donor normozoospermic samples, which were exposed ex vivo during four hours to a wireless internetconnected laptop showed a significant decrease in progressive sperm motility and an increase in sperm DNA fragmentation. These results showed a nonthermal negative effect on sperm, thus the researchers advised against locating a laptop near the testes.

### Other

#### Medical treatment factors

Antidepressants, tranquilizers, anti-hypertensives, medication for psoriasis, diabetes, rheumatism, gastric problems, seizure disorders, bowel disease, erectile dysfunction, and viral disease may all affect male fertility and should be critically evaluated and alternatives considered. A recent compilation can be found in Anderson, Nisenblat, Norman, 2010 (70). Again, it is important to note that even total medication-induced infertility has been shown to be reversible (71). A well designed study (n=165) by Hayashi et al. (72) has found that, in infertile men who were medicated with commonly used non-related drugs the semen quality improvement rate (93%) and conception rate (85%) were much higher in the study group that stopped or changed their medication when compared with the control group who had a 12% semen quality improvement rate and 10% conception rate. After changing medical treatments, the time interval before conception was 7.3 months in oligozoospermia and significantly shorter in asthenozoospermia.

#### Psychological conditions

Psychological distress and trauma, mood disorders, inadequate coping abilities, in addition to other psychological conditions may affect male fertility both psychologically (lack of motivation) and biologically (as a consequence of medical treatment) (73). A 2011 meta analysis of 57 crosssectional multinational studies (n=29914) showed that psychological stress could lower sperm density and sperm progressive motility and increase abnormal sperm (74). Endocrinologic processes are highlighted as the likely cause for reduced fertility on account of stress. Circulating levels of glucocorticoids increase stress and affect gonadal function at multiple levels in the hypothalamopituitary- gonadal axis by decreasing the synthesis and release of gonadotropin-releasing hormone (GnRH) in the hypothalamus, inhibiting synthesis and release of LH and FSH in the pituitary gland and directly modulating steroidogenesis and/or gametogenesis in the testes or ovaries (75).

Psychological treatment of mood disorders and psychosocial problems appears comparable in efficacy to medication and the preferred treatment when fertility is at stake (76).

#### Healthy habits and male infertility

Being healthy does not imply being fertile. Even the healthiest of men can suffer fertility problems at any moment in life. Unsuspected outside and inside factors have been shown to play a decisive role. Evidence of the negative effects on fertility of some outside factors, such as smoking, is conclusive. Other factors, although identified by initial evidence, are still issues of debate. As recently as 2008, a questionnaire study *cum* opinion article considered “healthy habits” to be unrelated to improving fertility, and stated "falsely believing that not engaging in unhealthy habits actually increases health". (77). However, a well-designed 2011 study in a university outpatient clinic in the Netherlands has shown that tailored preconception counseling about unhealthy dietary and lifestyle behaviors of subfertile couples had a positive effect on reproductive performance and pregnancy outcome (78).

**Table 1:** Lifestyle, environmental and psychological factors affecting male fertility


Exogenous negative factors	Reversible?	Treatment (in order of recommendation)

**Tobacco smoke**	Partly (3 months)	Self-help Therapy* Medication
**Alcohol**	Often (3 months)	Self-help Therapy Medication
**Marijuana**	Often (3 months)	Self-help Therapy
**Cocaine**	Often (1-3 months)	Self-help Therapy Medication
**Endocrine disruptive substance (EDS)**	Often	Self-help
**Hormones**	Often (1-3 months)	Self-help
**Scrotal heat exposure (?)**	Possibly	Self-help
**Medication**	Often (1-7 months)	Expert advice Self-help

Endogenous factors	Reversible?	Treatment

**BMI**	Yes (3-9 months)	Therapy Self-help Surgical
**Psychological stress**	Yes	Self-help Therapy
**Other mental disorders**	Often	Therapy Medication

Healthy habits	Duration of positive effects	Relevance prior to ART

**No tobacco**	± one sperm cycle	High
**Low alcohol versus addiction**	long term	High
**No recreative drugs**	± one sperm cycle	High
**Antioxidant-rich diet**	± one sperm cycle	High
**Moderate exercise**	± one sperm cycle	Medium


* Therapy; Counseling or psychotherapy.

The present study results suggest that no generally applicable “safe” and “unsafe” thresholds can be established for lifestyle, environmental and psychological factors which have proven to possibly be negative for infertility, making individual analysis and guidance essential.

## Conclusion

Male factor infertility and subfertility are clinical concepts that do not necessarily reflect an unchangeable situation. A considerable, growing body of evidence indicates that male fertility is co-determined by lifestyle, environmental and psychological factors whose negative influences, to a considerable extent, can be reversed or halted. Thus, early systemized efforts to detect and treat any negative influences on male fertility from lifestyle, the environment and psychological conditions could provide better chances for natural conception and enhanced success rates for ART. Efforts to improve male fertility may lead to delaying assisted reproductive treatment in justified cases and for relatively short periods. Controlled trials as to the effectiveness of a systemized application of such a preliminary step are needed.
